# Tattoos: risks and complications, clinical and histopathological approach^☆^

**DOI:** 10.1016/j.abd.2023.07.004

**Published:** 2024-03-22

**Authors:** David Chalarca-Cañas, Mario A. Caviedes-Cleves, Luis A. Correa-Londoño, Juan Pablo Ospina-Gómez, Margarita M. Velásquez-Lopera

**Affiliations:** a Department of Dermatology, School of Medicine, University of Antioquia, Medellín, Colombia; b Department of Pathology, School of Medicine, University of Antioquia, Medellín, Colombia

**Keywords:** Complications, Inks, Punctures, Skin, Tattooing

## Abstract

**Background:**

Skin modification through tattoos is as old as humanity itself. However, this trend is on the rise, and with the use of different types of pigments and application practices, both cutaneous and systemic complications can arise. Adverse reactions can be grouped into five classes: inflammatory, infectious, neoplastic, aesthetic, and miscellaneous. On histopathology, inflammatory reactions can exhibit a lichenoid pattern or present as spongiotic dermatitis, granulomatous reactions, pseudolymphoma, pseudoepitheliomatous hyperplasia, or scleroderma/morphea-like changes. This article reviews tattoo complications, including their clinical and histopathological characteristics.

**Methods:**

An open search was conducted on PubMed using the terms “tattoo”, “complications”, and “skin”. No limits were set for period, language, or publication type of the articles.

**Results:**

Reactions to tattoos are reported in up to 67% of people who get tattooed, with papulonodular and granulomatous reactions being the most common. Some neoplastic complications have been described, but their causality is still debated. Any pigment can cause adverse reactions, although red ink is more frequently associated with them. Patients with pre-existing dermatoses may experience exacerbation or complications of their diseases when getting tattoos; therefore, this procedure is not recommended for this patient group.

**Conclusions:**

Dermatological consultation is recommended before getting a tattoo, as well as a histopathological examination in case of complications. In patients who develop cutaneous inflammatory reactions following tattooing, additional studies are recommended to investigate systemic diseases such as sarcoidosis, pyoderma gangrenosum, atopic dermatitis, and neoplasms. It is important for physicians to be trained in providing appropriate care in case of complications.

## Introduction

### Definition

Permanent tattooing is a practice carried out for aesthetic purposes that involves modifying the color of the skin. There are different types of cosmetic tattoos, which are applied through micropigmentation in various areas of the body, such as the eyes, cheeks, lips, and eyebrows, among others. Their objective may be to simulate makeup or camouflage dermatological conditions such as vitiligo, alopecia, and surgical scars. Additionally, three-dimensional tattooing of the areola and nipple is also used as the final stage in reconstructive breast surgery.^1^, ^2^, ^3^

### History

The practice of tattooing has a long history dating back over 5000 years, and the term comes from the Tahitian word “ta-tau”, which means “the results of tapping”. The earliest evidence of tattooing was found on Ötzi, the iceman discovered in the Italian-Austrian Alps in 1991, who had over 50 charcoal tattoos on his arthritic joints. It is believed that the earliest tattoos had therapeutic purposes.^4^ Although the practice spread throughout Europe, it was prohibited by Emperor Constantine with the arrival of Christianity, and other religions such as Judaism and Islam did not accept it either. However, during oceanic expeditions in the 17^th^ century, tattooing was reintroduced to Western civilization and became a symbol of wealth among the upper classes in the 19^th^ century. C.H. Fellows is considered one of the first professional tattoo artists, and it was in 1870 in New York that the first tattoo studio was opened by Martin Hildebrant, a German immigrant. His major competitor was Samuel O'Reilly, who invented the tattoo machine in 1891, inspired by a device created by Thomas Edison. By around 1900, tattoo studios existed in almost every major city. At that time, tattoos were mainly used by bohemians from the underworld and circus artists and remained dormant to the public until the 1970s when tattooing became increasingly accepted by both sexes and all ages. Nowadays, it has become a fashion trend and is done for decorative purposes, enjoying great popularity in various cultures and age groups. Despite improvements in hygiene and sterilization practices, the reporting of adverse reactions is increasing due to the use of other components in tattoo ink.^5^

## Methods

An open search was conducted on PubMed without restrictions on date, language, or publication type to gather information on the dermatological complications of tattoos. The search terms used were “tattoo”, “complications”, and “skin”. Articles dealing with systemic complications related to tattoos were excluded. Additionally, some articles and local sources of information from Latin America and Colombia were reviewed. Some clinical and histopathological images of tattoo-associated skin complications were included, obtained from a Dermatology Department in Colombia with prior patient consent.

## Results and discussion

In the initial PubMed search using the term “tattoo”, 6,359 results were found. However, when using more specific search terms like “tattoo”, “complications”, and “skin”, the article set was limited to 391. Furthermore, some references to articles and local information from Latin America and Colombia on tattoo complications were incorporated. After reviewing the articles, 80 of them met the objectives of the search and were included in the review.

### Epidemiology

The practice of tattoos mainly occurs in adolescents and young adults but has gained popularity across all age groups. The prevalence of tattoos varies significantly among different regions of the world, and there is no official data indicating the percentage of the global population with tattoos. According to surveys conducted by statistics portals like Statista, approximately 30% of Americans have tattoos. In Colombia, a polymeric survey conducted by the portal Cifras y Conceptos in 2020 revealed that 6% of the population over 35 years old has tattoos, while 19% of Colombians between 25 and 34 years old and 47% of people between 18 and 24 years old have tattoos. It has been found that up to 23% of patients who get a tattoo are not satisfied with the result.^1^

With the increase in tattooing practice, there is a growing number of adverse reactions, which are often seen by doctors but are less known to the public. Usually, these adverse reactions do not endanger life but can significantly affect the quality of life and cause disfigurement. Some studies describe that up to 2.1% of patients who get tattoos experience some type of complication,^6^ but it is believed that the actual rate may be higher due to underdiagnosis, as many people do not seek medical advice or self-medicate. In fact, some studies have reported rates as high as 67% of adverse skin reactions.^7^ The most used color in tattoos is black, but adverse reactions more commonly occur in response to red ink. Green ink is less frequently implicated in tattoo reactions, but it has been reported to worsen cutaneous reactions in patients during patch testing.^5^

### Regulation in Colombia and the world

Although the pigments used in tattoo inks must be approved by the Federal Food, Drug, and Cosmetic Act before being marketed, the Food and Drug Administration (FDA) has not regulated their use due to the priority of other public health issues and the lack of evidence of specific safety problems related to these pigments. The FDA classifies tattoo inks as cosmetics and takes measures to prevent safety issues and evaluate the severity of adverse events and the safety of pigments in case safety problems are identified.

In Colombia, the regulation of tattooing practice follows the same requirements as cosmetic establishments, according to Resolution 2263 of 2004 by the Ministry of Health of Colombia. The regulation is limited to opening, operation, biosafety, and training requirements but does not extend to the surveillance of adverse events related to tattoo inks.^8^, ^9^

### Tattoo ink

Inks are suspensions that contain metallic salts and organic compounds in a liquid vehicle such as water, alcohol, and glycerin. In the past, inks were rich in metallic salts, such as mercury sulfide, which was responsible for many adverse reactions. Currently, these components are still present but in smaller amounts. The components vary depending on the color of the ink. Black ink contains zinc oxide and carbon; white ink contains lead carbonate, titanium dioxide, barium sulfate, and zinc oxide; red ink contains mercury sulfide (cinnabar), cadmium selenide (cadmium red), sienna earth, ferric hydrate (red ochre), and organic dyes; brown ink contains iron oxide (ochre); yellow ink contains cadmium yellow, ochre, turmeric yellow, and chrome yellow; green ink contains chromium oxide, lead chromate, phthalocyanine dyes, ferrocyanides, and ferricyanides; blue ink is composed of cobalt blue, cobalt phthalocyanine, and cobalt aluminate; purple ink contains ammonium and manganese pyrophosphate, aluminum salts, and dioxazine/carbazole.^10^, ^11^ Currently, many of these components are being replaced by azoic pigments (used in printers or automobile paint) because they are more colorful and resistant to fading.^12^ However, they may contain impurities such as aromatic amines.^13^ Antimony, cadmium, lead, chromium, cobalt, nickel, and arsenic may be present as contaminants.^14^

Inorganic metal salts or vegetable pigments mixed with cigarette ashes or other types of inks are used by inexperienced amateur tattoo artists, with a higher risk of adverse events.^15^ Most of the components used in permanent tattoos are toxic, mutagenic, or carcinogenic. As a result, adverse reactions can occur with a wide range of manifestations that may appear immediately or years after the tattoo placement.^16^ Superficial henna tattoos only dye the stratum corneum without introducing pigment into the dermis. These tattoos contain additives such as lemon juice or beet, walnut shell, sugar, and paraphenylenediamine, which accelerate the fixation process and improve the tattoo's duration, lasting 1‒3 weeks.^17^, ^18^

### Pathophysiological mechanism

The mechanism involves the disruption of the cutaneous barrier through the repetitive application of needles manually or by electric machines, followed by the deposition of exogenous colored particles below the dermo-epidermal junction at a depth of 1‒3 mm, reaching the papillary and reticular dermis. The rupture of superficial capillaries during the punctures causes mild bleeding that mixes with the ink. The colored particles are poorly soluble and highly resistant to enzymatic degradation, which perpetuates their presence in the dermis. The disruption of the basal membrane and necrosis of some dermal and epidermal cells leads to an acute inflammatory response during the first two hours of the procedure, where polymorphonuclear cells phagocytize the pigment. This clinically manifests as transient edema. At 24 hours, the pigment accumulates in the cytoplasmic phagosomes of keratinocytes, histiocytes, mast cells, and fibroblasts. During the first month of the procedure, before the regeneration of the basal membrane, part of the pigment is eliminated transepidermally and can be found in keratinocytes, macrophages, and fibroblasts. During this healing phase, some pathogenic microorganisms can infect the skin. Once the basal membrane has regenerated, no further transepidermal elimination occurs, reducing the risk of infectious complications. After the host's acute response, a reaction to the foreign material (ink) occurs, initially involving a cell-mediated immune response, histologically characterized by a dense lymphocytic inflammatory infiltrate in the dermis. Larger particles do not transport to other anatomical sites and become residents of the dermis, where they are sequestered by mononuclear cells, fibroblasts, and extracellular tissue. Smaller ink particles penetrate deeper into the dermis and are recognized by Langerhans cells, which eventually transport them to the lymph nodes, generating a reactive lymphocytic response. Clinically, this presents as lymphadenopathy, and histologically, it is represented by the presence of pigment in the lymph nodes with abundant histiocytes and Langerhans cells in the paracortex (dermatopathic lymphadenopathy).^5^, ^19^, ^20^ Exaggerated or dysregulated responses, as well as the presence of toxic substances, generate the complications mentioned below.

### Clinical manifestations

Adverse cutaneous reactions can occur in various forms, including edema, allergic reactions, itching, local pain, erythema, bleeding, papule formation, nodules, intense pain, and, rarely, neoplasms. Additionally, temporary henna-based tattoos can cause acute eczematous reactions that progress to depigmentation, hypertrophic scars, or more severe lichenoid reactions.^21^ Categorically, these reactions can be divided into five groups: inflammatory, infectious, neoplastic, aesthetic, and miscellaneous. Inflammatory reactions are further histopathologically categorized into a lichenoid pattern, spongiotic dermatitis, granulomatous pattern, pseudolymphoma, pseudoepitheliomatous hyperplasia, and sclerodermiform/morpheaform pattern, among others.^16^ In a systematic review, it was found that among all complications associated with tattoos, granulomatous reactions accounted for 48.48%, infectious reactions for 21.21%, and allergic reactions for 12.12%.^22^

### Inflammatory complications

#### Transient edema

Transient edema is an inevitable reaction, and it is considered that up to a third of tattooed individuals may experience this minor complication.^23^ It manifests as induration of the tattooed area and may or may not be accompanied by pruritus, disorders in skin pigmentation, and focal dermal hemorrhagic disorders, among others, which self-resolve within a period of 2 to 3 weeks after pigment injection. These manifestations may result from the trauma of hundreds of needle punctures, irritation from alcohol use, or an acute inflammatory reaction to the ink and/or diluent, leading to disruption of the epidermal basal membrane and some necrosis of dermal and epidermal cells.^24^, ^25^

#### Allergic/Immune-mediated reactions

Allergic contact dermatitis is characterized by eczematous, hyperkeratotic, or ulcer-necrotic lesions in the tattooed area, which may be photosensitized due to alteration of pigments through Ultraviolet (UV) decomposition or haptenization of antigens. Haptenization occurs when UV light affects the pigments and alters their chemical composition, making them recognizable as foreign substances and triggering an immune response. This can result in erythematous, vesicular, or exfoliative eruptions.^26^ Despite the transition to azoic-based inks, hypersensitivity reactions in tattoos are still primarily attributed to the presence of mercury in red inks.^16^, ^27^ Regarding other ink colors, confirming the responsible allergen is difficult to achieve through patch testing due to the lack of precise lists of ingredients and comparable allergens for testing, and the results are often negative.^28^ This reinforces the idea that ink haptenization is a necessary step in the pathogenesis of adverse reactions.^28^ It is important to note that the main differential diagnosis for this reaction is pseudolymphoma, which often affects more than one color of the tattoo.^16^

#### Chronic skin diseases

Tattoos can trigger the appearance of new lesions in individuals with preexisting dermatoses. These lesions typically appear a few days to weeks after the tattoo, although they can also extend for several months.^29^ Chronic skin diseases such as psoriasis, vitiligo, granuloma annulare, lichen planus, lichen sclerosus, pyoderma gangrenosum, lupus erythematosus, morphea, and Darier's disease share a common characteristic: cutaneous manifestations can be reproduced in areas of focal trauma (Koebner's isomorphic phenomenon).^5^, ^27^ Patients with atopic dermatitis may develop contact allergy and photosensitivity reactions that trigger eczematous reactions or worsen exacerbations.^27^ Similarly, patients with preexisting pyoderma gangrenosum have been reported to develop ulcers resembling this condition at the tattoo site, especially in the lower extremities, due to the pathergy phenomenon.^30^, ^31^ It is important to consider the recommendation that patients with preexisting dermatoses abstain from getting tattoos or be evaluated by a dermatologist before doing so, due to the potential risk of exacerbating the underlying pathology.

#### Papulonodular reactions

**Figure 1:**
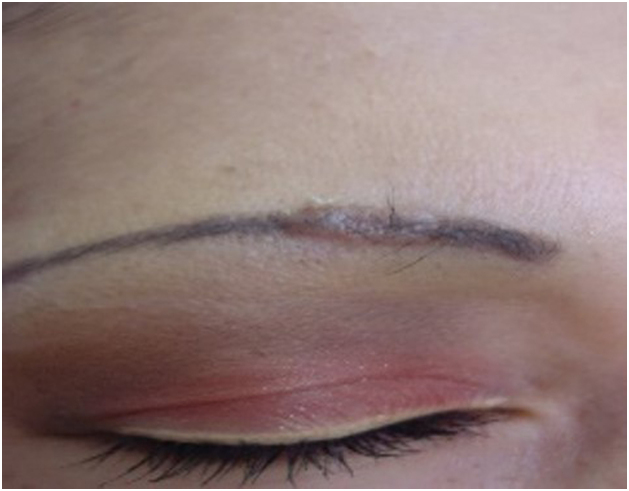
Papulonodular lesions following cosmetic eyebrow tattooing. The final diagnosis was sarcoidosis. Photographic archive of the Dermatology Service at the University of Antioquia.

**Figure 2:**
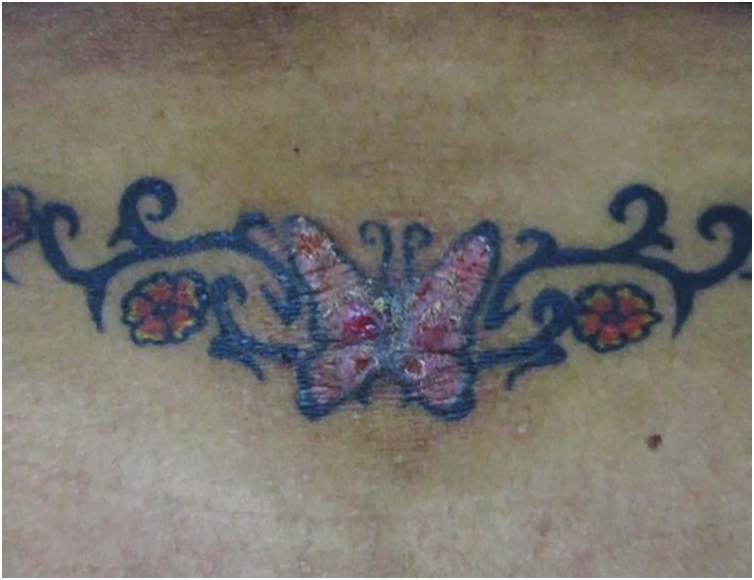
Papulonodular lesions over a black and red tattoo in the lower lumbar region. The histopathological diagnosis was pseudolymphoma. Photographic archive of the Dermatology Service at the University of Antioquia.

These occur months or years after the tattoo and are primarily associated with foreign body-type reactions, with approximately 29.3% of cases being associated with cutaneous or systemic sarcoidosis (Fig. 1). Granulomatous skin reactions associated with tattoos can be an initial presentation of a systemic disease like sarcoidosis, requiring additional studies such as chest radiography or computed tomography and laboratory tests.^32^ The mechanism of sarcoidosis induction in individuals with tattoos could be pigment overload, which can occur at the edges or corners of a tattoo where pigment density is higher than in other areas, or in cases where there is repeated introduction of foreign material. This could lead to chronic immune system exposure to tattoo ink, resulting in sustained foreign body-type granulomatous inflammation and subsequent development of focal autoimmunity in genetically susceptible individuals for sarcoidosis development. This explains the often-prolonged latency period between tattoo placement and the appearance of clinical symptoms.^33^, ^34^ Sarcoidosis has been detected in tattoos of patients who have been treated with interferon for any reason. For this reason, it is suggested that individuals with a history of sarcoidosis avoid tattooing if they are being treated with interferon. Asymptomatic or pruritic indurated erythematous-violet papules and nodules on the tattooed area (Fig. 2) can also occur secondary to lichen planus, pseudolymphomas, lymphomatoid papulosis, lymphomas, among others, with the diagnosis confirmed through histopathology.^35^

#### Vasculitis

The relationship is not clear; however, isolated cases of hypersensitivity vasculitis have been reported. It is important to rule out other causes of vasculitis such as infection, medications, malignancy, or systemic diseases.^36^

### Histopathological classification of inflammatory complications from tattoos

#### Lichenoid inflammatory pattern

**Figure 3:**
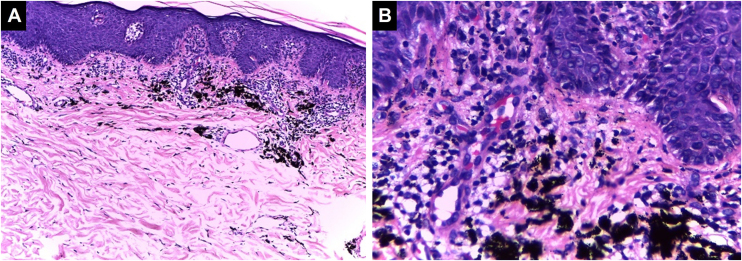
**Lichenoid pattern.** Epidermal hyperplasia, orthokeratosis, Malpighian layer with spongiosis and scattered dyskeratocytes. Vacuolar damage of the basal layer. In the dermis, there is an inflammatory infiltrate in a band-like pattern with some cytoid bodies containing black pigment, (Hematoxylin & eosin, [A] ×100, [B] ×400). Photographic archive of the Dermatopathology Laboratory, Dermatology Section, University of Antioquia.

It is the most common hypersensitivity tissue reaction that can occur in response to any ink color, but with a predilection for red ink due to the presence of components such as nickel, mercury, and cadmium, presenting even as verrucous lesions.^37^ Lichenoid reactions can be caused by lichen planus or overlap with allergic reactions, and sometimes it is difficult to distinguish between these two etiologies, making clinical correlation crucial. Sometimes the lichenoid inflammatory pattern is associated with the Koebner isomorphic phenomenon in patients with undiagnosed lichen planus, so when it is found, other anatomical sites should be examined.^38^, ^39^ Histopathologically, it is characterized by a band-like lymphocytic dermal inflammatory infiltrate with interface changes, basal vacuolar degeneration, and dyskeratotic keratinocytes^40^ (Fig. 3).

#### Spongiotic dermatitis

**Figure 4:**
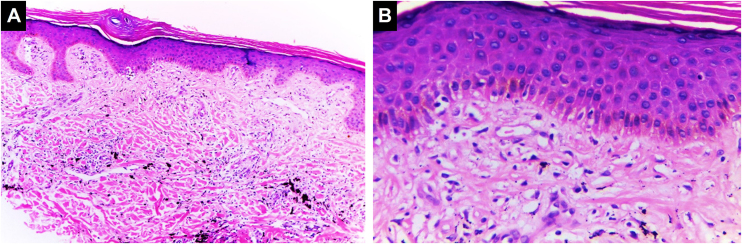
**Spongiotic dermatitis.** Preserved thickness of the epidermis with marked spongiosis and some dyskeratocytes. Basal vacuolar damage. Black ink pigment is identified in the dermis, (Hematoxylin & eosin, [A] ×100, [B] ×400). Photographic archive of the Dermatopathology Laboratory, Dermatology Section, University of Antioquia.

This reaction can be found in the acute presentation following the tattoo (transient edema) or can be the manifestation of an allergic response. Histopathologically, it is characterized by intercellular epidermal edema (eczema) accompanied by a mixed or non-mixed chronic lymphocytic dermal inflammatory infiltrate, which can overlap with the lichenoid pattern 41, 42 (Fig. 4).

#### Granulomatous inflammatory pattern

**Figure 5:**
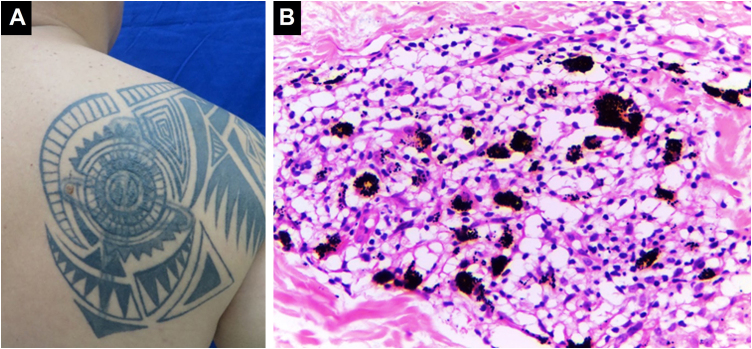
**Granulomatous inflammation of a sarcoid type.** (A) Papulonodular lesions in a tattoo on the right scapular region. (B) Granulomatous infiltrate of sarcoid type associated with black ink pigment (Hematoxylin & eosin, ×400). Systemic studies revealed sarcoidosis. Photographic archive of the Dermatopathology Laboratory, Dermatology Section, University of Antioquia.

An inflammatory infiltrate composed of epithelioid histiocytes, multinucleated giant cells with variable lymphocytic and polymorphonuclear infiltrate, which can organize into different patterns, of which foreign body granuloma and sarcoidal granuloma are the most common. Foreign body granulomas are characterized by pigment-laden foreign body-type giant cells. Sarcoidosis is defined by non-caseating epithelioid granulomas with little or no accompanying inflammatory response^43^ (Fig. 5). Staining and/or cultures should be performed to rule out fungal or mycobacterial infection. Granulomas with central necrosis (tuberculoid) are mainly observed in tuberculosis and leprosy, although they have also been described in response to ferric oxide and chromium salts in cosmetic tattoos. Granuloma annulare and necrobiosis lipoidica-like reactions are rarer.^16^, ^27^, ^38^ In one described case, a disease similar to Rosai-Dorfman was observed with a dense lymphocytic and histiocytic inflammatory infiltrate in the dermis. Abundant histiocytes with emperipolesis of inflammatory cells were found. However, unlike Rosai-Dorfman disease, there was no involvement of lymph nodes, which is a characteristic finding of that disease.^44^

#### Pseudolymphoma

Pseudolymphoma is a clinically benign reaction characterized histopathologically by dense nodular dermal lymphocytic infiltrates or band-like lichenoid infiltrates. Immunohistochemical studies may show the presence of B- and T-cells with a normal immunophenotype, and gene rearrangement studies will reveal polyclonal lymphocyte populations.^45^ The pathogenesis is uncertain; however, it has been reported to occur frequently in areas of tattoos with red ink, which induces chronic antigenic stimulation resulting in polyclonal proliferation of B- and T-lymphoid cells. Although malignant transformation is rare, cases of B-cell lymphomas associated with tattoos have been described, so close follow-up is recommended. The differential diagnosis includes allergic reactions, B-cell and T-cell lymphomas, lymphomatoid papulosis, and infections.^46^, ^47^

#### Pseudoepitheliomatous hyperplasia

It is a reactive pattern characterized by marked epidermal hyperplasia accompanied by follicular hyperkeratosis and variable dermal inflammation composed of lymphocytes and plasma cells.^48^ It can be triggered by stimuli such as chronic irritation, scarring, trauma, and nonspecific inflammatory or infectious dermatoses, presenting between 2 weeks to 3 months after tattooing and should be differentiated from neoplastic entities such as keratoacanthoma and squamous cell carcinoma.^49^

#### Scleroderma/Morphea-like pattern

**Figure 6:**
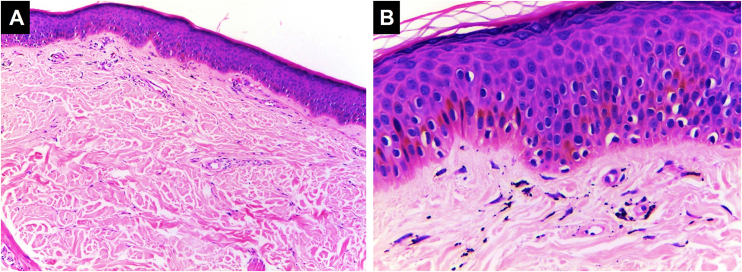
**Sclerodermiform/morphea-like pattern.** The epidermis appears unaltered in thickness. In the dermis, fibrosis with effacement of cutaneous appendages is observed, associated with deposition of black ink pigment, (Hematoxylin & eosin, [A] ×100, [B] ×400). Photographic archive of the Dermatopathology Laboratory, Dermatology Section, University of Antioquia.

Characterized by dermal sclerosis and/or dermal fibrosis, which can be secondary to an underlying connective tissue disease or represent the development of dermal fibrosis in a setting of chronic inflammation or hypersensitivity^50^ (Fig. 6).

Other described histopathological patterns include psoriasiform, vasculitic, and neutrophilic dermatosis.

### Infectious complications

Due to the disruption of the skin's physical and immune barrier and the alteration of the cutaneous ecosystem, tattoos facilitate the inoculation of pathogenic microorganisms into the dermis, increasing the risk of infectious complications, especially in immunosuppressed patients. In informal settings with poor hygiene, the risk is higher. Infections can be of bacterial, viral, fungal, mycobacterial, and, more rarely, parasitic origin. Because of this, in some countries, according to local laws and regulations, individuals with tattoos are prohibited from donating blood for periods ranging from 4 months to 1 year after getting a tattoo. In Colombia, the minimum time is 6 months.^51^ When the technique is performed by an experienced professional tattoo artist using hygienic and sterile techniques, on healthy skin with proper post-procedure care, the risk is very low.^6^

#### Bacterial infections

Bacterial infectious complications have been estimated to affect 1%‒5% of tattooed individuals.^14^ The most frequently reported ones include impetigo, folliculitis,^52^ furunculosis, abscesses, ecthyma, cellulitis, erysipelas, and gangrene, with the main causative agents being *Staphylococcus aureus*, *Streptococcus pyogenes*, *Clostridium difficile*, and *Pseudomonas aeruginosa*.^53^ Clinical manifestations typically appear within days to a few weeks after getting the tattoo and may include local pain, erythema, edema, fever, and, in some cases, purulent discharge. Some studies have reported that up to 28% of tattoo ink bottles are improperly sealed, and 10% may be contaminated with both pathogenic and non-pathogenic bacteria, posing a significant risk of adverse reactions to this practice.^54^ Transmission of syphilis through tattoos is anecdotal and was described in the past because of contamination with saliva from syphilitic tattoo artists. There have also been reported cases of secondary syphilis in tattooed areas, although these are currently rare.^55^, ^56^ Generally, bacterial infections are easily treatable, and the treatment does not significantly differ from that of other infectious conditions.^16^

#### Mycobacterial infections

Non-tuberculous mycobacteria are ubiquitous in water and can infect tattoo inks when gray ink is prepared by diluting black ink with non-sterile or non-distilled tap water. Additionally, ink dilution reduces the antimicrobial efficacy of any preservatives that may be present.^57^ The most frequently isolated mycobacteria include *Mycobacterium chelonae*, *Mycobacterium haemophilum*, *Mycobacterium abscessus*, *Mycobacterium immunogenum, Mycobacterium massiliense*, and *Mycobacterium fortuitum*.^58^ Clinically, papules, pustules, or ulcerated nodules limited to the tattooed area may be observed.^59^

In areas where leprosy is endemic, cutaneous inoculation with *Mycobacterium leprae* can occur, and manifestations may appear decades after the inoculation.^60^ In 2002, Ghorpade et al. described 31 cases of leprosy inoculated through tattoos and established the following diagnostic criteria: 1) Appearance of the first leprosy lesion after the tattoo, on the same site; 2) Presence of a single leprosy lesion on a single tattoo mark; 3) Histopathological evidence of leprosy and dermal tattoo pigment; 4) Absence of any pre-existing skin disease before the tattoo; 5) Demonstration of *M. leprae* in the tattoo instrument through smears or inoculation in the footpad of a mouse, if possible.^60^

Infections by *Mycobacterium tuberculosis* or *Mycobacterium bovis* have also been reported after tattooing, with clinical manifestations appearing 2–4 weeks after inoculation. They are characterized by the formation of papules or erythematous nodules that progress to superficial ulcers (tuberculous chancre), accompanied by painless regional lymphadenopathy. In immunocompromised patients, progression to lupus vulgaris and tuberculosis cutis verrucosa, or even hematogenous dissemination, may occur.^6^, ^61^

These infections should be considered when patients present with skin reactions decades after getting a tattoo or in patients who have been tattooed in prison or in environments where sterile techniques are not employed. Diagnostic tests include biopsy, tissue culture, and PCR for mycobacteria. Special stains for mycobacteria in biopsies are useful when positive but are not sufficient to rule out an infection. The tuberculin skin test also has great diagnostic value.^16^, ^27^

#### Viral infections

Some viral infections can be transmitted through contaminated instruments and inks used during tattooing. The most described ones include HIV, Hepatitis B (HBV), and Hepatitis C (HCV), although the risk of transmission under optimal biosafety conditions is very low.^5^, ^62^ There have been reports of warts appearing at tattoo sites, with some cases being triggered by intense sunburn. It was postulated that local immunosuppression induced by UV radiation may reactivate the Human Papillomavirus (HPV). Therefore, a reasonable recommendation is to protect freshly tattooed skin from UV exposure.^27^, ^63^ The most common serotypes of HPV are 3, 10, 27, 28, 47, and 49.^23^ Infection by the molluscum contagiosum virus may occur weeks or months after tattooing, and when it becomes widespread, it can happen in the context of an HIV infection.^27^ Herpes simplex virus type 1 can cause primary infection during the procedure or during the healing phase. In patients with a history of HSV-1 infection, it can reactivate, known as “herpes compunctorum”. ^64^ Other viral infections possibly related to tattooing are molluscum contagiosum, rubella, and vaccinia. It should be noted that several of these infectious conditions are declining due to vaccination practices in early life stages and the increased use of aseptic techniques that mitigate the risk of viral transmission. 5, 65

#### Fungal infections

Cutaneous fungal infections in tattooed areas are very rare, although some cases of ringworm, pityriasis versicolor, candidiasis, sporotrichosis, aspergillosis, zygomycosis, or Acremonium infections have been described.^27^, ^59^ Superficial mycoses can occur during the tattoo healing process, with the main etiological agent being *Microsporum canis*, a dermatophyte isolated from the hair of domestic animals.^23^

#### Parasitic infection

These are extremely rare. Some cases of cutaneous leishmaniasis have been reported after tattoos in individuals with visceral leishmaniasis or HIV.^27^

### Neoplastic complications

**Figure 7:**
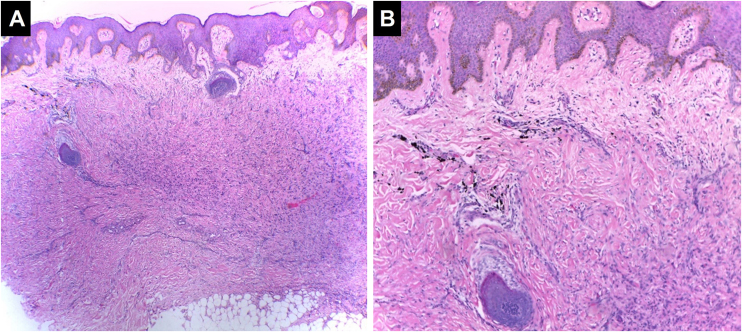
**Dermatofibroma.** Dermis with non-encapsulated neoplasm of spindle cells with mild cytological atypia, surrounded by black ink pigment. The epidermis shows pseudoepitheliomatous hyperplasia, (Hematoxylin & eosin, [A] ×40, [B] ×100). Photographic archive of the Dermatopathology Laboratory, Dermatology Section, University of Antioquia.

Some cutaneous neoplasms have been described in association with tattooing, although there is limited epidemiological data to support this causal relationship. Benign neoplasms that have been reported include dermatofibromas (Fig. 7), seborrheic keratosis, epidermal cysts, miliaria, and pseudoepitheliomatous hyperplasia. However, it is believed that these neoplasms are underreported as they are rarely published.^49^, ^66^ Other neoplasms more frequently reported include melanoma,^67^ basal cell carcinoma,^68^ squamous cell carcinoma,^69^ and keratoacanthoma.^70^, ^71^ Rare cases of dermatofibrosarcoma protuberans, cutaneous leiomyosarcoma, and cutaneous lymphoma have also been reported.^72^ Melanoma and basal cell carcinomas have been primarily reported in black, dark blue, or other dark-colored tattoos, while squamous cell carcinomas, keratoacanthomas, and pseudoepitheliomatous hyperplasia have been more frequently associated with red tattoos.^72^ The pathogenesis could be multifactorial, given the presence of carcinogenic substances in tattoo ink such as aromatic amines and polycyclic aromatic hydrocarbons;^14^ the trauma induced by the tattooing procedure; chronic inflammatory response to foreign material in the skin; UV radiation; and genetic predisposition. Additionally, laser tattoo removal to break down the pigment into smaller segments could release even more carcinogens that may be present in the host's reticuloendothelial system. Furthermore, it is believed that when a tattoo is applied over a nevus, the trauma and carcinogenic substances could induce dysplasia.^72^ While these findings may be coincidental, some case series have demonstrated the occurrence of neoplasms in tattoos at a younger age compared to epidemiological data in the general population, warranting further studies to establish this association.^71^, ^73^ However, it is advisable for patients with melanocytic lesions or risk factors for melanoma to refrain from tattooing over pigmented lesions, and it would be ideal for tattoo artists to be aware of these complications.

### Cosmetic complications

Dissatisfaction with the tattoo is the most common cosmetic complication, as it can distort the appearance of normal skin. Injecting pigment too deeply into the subcutaneous fat can cause pigment migration into the surrounding skin outside the edges of the original tattoo, resulting in distorted contours or a “blow-out” phenomenon, which can occur immediately after the completion of the tattoo.^74^, ^75^ Since a tattoo is considered a massive trauma to the skin, it can rarely induce the formation of hypertrophic scars and keloids.^76^

### Miscellaneous complications

#### Neurosensory complications

Pain and intense itching can occur after a tattoo and have been associated with possible involvement of cutaneous nerve branches, especially C fibers of sensory nerves. ^77^

**Cutaneous complications during Magnetic Resonance Imaging (MRI)**

During MRI scans, cutaneous manifestations can occur in the tattooed area, such as skin irritation, edema, and burning sensation. This could be caused by zinc oxide present in tattoo inks or by metals with ferromagnetic properties capable of conducting currents and heating adjacent tissues. 78, 79 Additionally, the metallic pigments in the inks can interfere with the quality of the MRI and cause artifacts in the image. It should be emphasized that tattoos are not a contraindication for undergoing MRI. ^16^

#### Photoinduced reactions

Reactions related to sunlight exposure have been found in up to 20% of cases. ^80^ Clinically, they manifest as erythematous and edematous lesions, primarily described in tattoos containing cadmium sulfide, which is usually present in traditional yellow and red pigments. ^6^ The mechanism is believed to involve the induction of reactive species that interact with DNA, proteins, or lipids, compromising their normal function and mediating symptoms such as pain, itching, or cell death. ^81^

### Treatment

Tattoo removal is a laborious, potentially unsatisfactory, and problematic process. The treatment modality depends on the severity and location of the lesions. Therapeutic approaches for localized cutaneous lesions primarily involve the application of topical corticosteroids, intralesional and systemic corticosteroids, and avoiding exposure to ultraviolet light. Oral treatment with antihistamines may be useful in cases of symptomatic acute complications. In severe cases, dermabrasion, cryotherapy, surgical excision, chemical destruction, and laser ablation can be employed. However, the treatment should be directed towards addressing the underlying or de novo pathology that arises after tattoo application. In infectious complications, the treatment is aimed at the causative agent. In the case of molluscum contagiosum, spontaneous involution may occur; otherwise, removal with a curette or fine needle is well tolerated. Warts can be treated with cryosurgery, electrosurgery, and laser; topical treatment with keratolytics (salicylic acid and topical retinoids), imiquimod, 5-fluorouracil, photodynamic therapy, and potassium hydroxide are also valid options. Superficial fungal infections can be treated with terbinafine and topical or systemic imidazoles, depending on the severity of the condition. In the presence of bacterial infections, it is important to administer targeted antibiotic treatment based on the specific entity and the isolated pathogen in cultures.^22^, ^23^ Photo-induced reactions are treated like any other phototoxic reaction, and preventive measures such as covering tattoos from ultraviolet light or using sunscreen are recommended.^16^

## Conclusions

Tattoos are a highly popular practice but are poorly regulated, increasing the risk of adverse reactions. Although rare, these reactions can cause discomfort and damage the appearance of the skin. Red ink is most associated with adverse reactions, but any color can cause them. Patients are advised to consult with a dermatologist before getting a tattoo, especially those with chronic dermatoses, as they may worsen after the procedure. Additionally, tattooing over scarred areas is not recommended as it could increase the risk of sarcoidosis development. Inflammatory reactions, especially granulomatous ones, are well-described. In the presence of cutaneous complications, further studies are recommended to investigate systemic diseases such as sarcoidosis. Patients who get tattoos should take sun protection measures. While a causal relationship between tattoos and benign and malignant neoplasms has not been established, many components of tattoo ink are carcinogenic, making neoplasms a serious complication associated with this practice. It is advisable not to tattoo over pre-existing nevi as it could increase the risk of developing melanoma. Therefore, skin biopsies are recommended for all cutaneous reactions to tattoos, as some may cause systemic complications. It is important for physicians to be familiar with the identification and treatment of short-term and long-term complications. Public awareness of the different types of tattoos and their complications is needed. The need for broader, well-designed studies to establish guidelines for regulating the use of inks and pigments is emphasized. In summary, it is essential for patients considering getting a tattoo to be well-informed about the potential risks associated with this practice, and medical professionals should be trained to provide appropriate care in case of complications.

## Financial support

None declared.

## Authors’ contributions

David Chalarca-Cañas: Final approval of the final version of the manuscript; critical review of the literature; data collection, analysis, and interpretation; writing of the manuscript or critical review of important intellectual content; effective participation in the research guidance; the study concept and design.

Mario A. Caviedes-Cleves: Final approval of the final version of the manuscript; critical review of the literature; data collection, analysis, and interpretation; writing of the manuscript or critical review of important intellectual content; effective participation in the research guidance; the study concept and design.

Luis A. Correa-Londoño: The study concept and design; data collection, or analysis, and interpretation of data; writing of the manuscript or critical review of important intellectual content; effective participation in the research guidance; final approval of the final version of the manuscript.

Juan Pablo Ospina-Gómez: The study concept and design; data collection, or analysis, and interpretation of data; writing of the manuscript or critical review of important intellectual content; effective participation in the research guidance; final approval of the final version of the manuscript.

Margarita M. Velásquez-Lopera: The study concept and design; data collection, or analysis and interpretation of data; writing of the manuscript or critical review of important intellectual content; effective participation in the research guidance; intellectual participation in the propaedeutic and/or therapeutic conduct of the studied cases; final approval of the final version of the manuscript.

## Conflicts of interest

None declared.
